# Disrupted Higher-Order Topology in OCD Brain Networks Revealed by Hodge Laplacian – an ENIGMA Study

**DOI:** 10.64898/2026.03.04.709586

**Published:** 2026-03-06

**Authors:** Hanyang Ruan, Moo K. Chung, Willem B. Bruin, Nadža Džinalija, Yoshinari Abe, Pino Alonso, Alan Anticevic, Srinivas Balachander, Marcelo C. Batistuzzo, Francesco Benedetti, Sara Bertolín, Silvia Brem, Youngsun Cho, Federica Colombo, Beatriz Couto, Goi Khia Eng, Sónia Ferreira, Jamie D. Feusner, Rachael G. Grazioplene, Patricia Gruner, Kristen Hagen, Bjarne Hansen, Yoshiyuki Hirano, Marcelo Q. Hoexter, Jonathan Ipser, Fern Jaspers-Fayer, Minah Kim, Jun Soo Kwon, Luisa Lazaro, Chiang-Shan R. Li, Christine Lochner, Rachel Marsh, Ignacio Martínez-Zalacaín, Jose M. Menchón, Pedro S. Moreira, Pedro Morgado, Emma Muñoz, Akiko Nakagawa, Janardhanan C. Narayanaswamy, Erika L. Nurmi, Joseph O’Neill, Jose C. Pariente, John C. Piacentini, Maria Picó-Pérez, Fabrizio Piras, Federica Piras, Christopher Pittenger, Janardhan Y. C. Reddy, Daniela Rodriguez-Manrique, Yuki Sakai, Joao R. Sato, Eiji Shimizu, Venkataram Shivakumar, Helen B. Simpson, Carles Soriano-Mas, Nuno Sousa, Emily R. Stern, Evelyn Stewart, Philip R. Szeszko, Sophia I. Thomopoulos, Anders L. Thorsen, Benedetta Vai, Anouk van der Straten, Ysbrand D. van der Werf, Wieke van Leeuwen, Hein van Marle, Guido van Wingen, Daniela Vecchio, Ganesan Venkatasubramanian, Chris Vriend, Susanne Walitza, Zhen Wang, Tokiko Yoshida, Je-Yeon Yun, Qing Zhao, ENIGMA OCD Working Group, Paul M. Thompson, Dan J. Stein, Odile A. van den Heuvel, Kathrin Koch

**Affiliations:** 1. Department of Neuroradiology, TUM University Hospital, School of Medicine and Health, Technical University of Munich (TUM), Munich, Germany,; 2. School of Medicine and Health, TUM-NIC Neuroimaging Center, Technical University of Munich, Munich, Germany,; 3. Department of Biostatistics and Medical Informatics, University of Wisconsin-Madison, Madison, WI, USA,; 4. Department of Anatomy & Neurosciences, Amsterdam University Medical Center, Amsterdam, the Netherlands,; 5. Department of Psychiatry, Amsterdam University Medical Center, Amsterdam, the Netherlands,; 6. Compulsivity, Impulsivity and Attention program, Amsterdam Neuroscience, Amsterdam, the Netherlands,; 7. Department of Psychiatry, Graduate School of Medical Science, Kyoto Prefectural University of Medicine, Kyoto, Japan,; 8. Sugimoto Psychiatric Clinic, Kyoto, Japan,; 9. Bellvitge Biomedical Research Insitute-IDIBELL, Bellvitge University Hospital, Barcelona, Spain,; 10. CIBERSAM, Instituto de Salud Carlos III, Madrid, Spain,; 11. Department of Clinical Science, Faculty of Medicine, University of Barcelona, Barcelona, Spain,; 12. Department of Psychiatry, Yale University, New Haven, CT, USA,; 13. OCD clinic, Department of Psychiatry, National Institute of Mental Health And Neurosciences (NIMHANS), Bangalore, India,; 14. Department of Psychiatry, University of Sao Paulo School of Medicine, Sao Paulo, Brazil,; 15. Department of Methods and Techniques in Psychology, Pontifical Catholic University, Sao Paulo, Brazil,; 16. Psychiatry & Clinical Psychobiology, Division of Neuroscience, IRCCS Scientific Institute Ospedale San Raffaele, Milano, Italy,; 17. Vita-Salute San Raffaele University, Milano, Italy,; 18. Department of Child and Adolescent Psychiatry and Psychotherapy, University Hospital of Psychiatry Zurich, University of Zurich, Switzerland,; 19. Neuroscience Center Zurich, University of Zurich and ETH Zurich, Switzerland,; 20. Life and Health Sciences Research Institute (ICVS), School of Medicine, University of Minho, Braga, Portugal,; 21. ICVS/3B’s, PT Government Associate Laboratory, Braga/Guimarães, Portugal; 22. Clinical Academic Center - Braga, Braga, Portugal,; 23. Department of Psychiatry, New York University Grossman School of Medicine, New York, NY, USA,; 24. Clinical Research, Nathan Kline Institute for Psychiatric Research, Orangeburg, NY, USA,; 25. Department of Psychiatry, Division of Neurosciences and Clinical Translation, University of Toronto, Toronto, ON, Canada,; 26. Centre for Addiction and Mental Health, Toronto, ON, Canada,; 27. Department of Women’s and Children’s Health, Karolinska Institutet, Stockholm, Sweden,; 28. Bergen Center for Brain Plasticity, Haukeland University Hospital, Bergen, Norway; 29. Molde Hospital, Møre og Romsdal Hospital Trust, Molde, Norway,; 30. Regional Centre for Child and Youth Mental Health and Child Welfare (RKBU), Department of Mental Health, Faculty of Medicine and Health Sciences, Norwegian University of Science and Technology (NTNU), Trondheim, Norway,; 31. Center for Crisis Psychology, University of Bergen, Bergen, Norway,; 32. Research Center for Child Mental Development, Chiba University, Chiba, Japan,; 33. United Graduate School of Child Development, The University of Osaka, Suita, Japan,; 34. Department of Psychiatry and Mental Health and Neuroscience Institute, Brain Behaviour Unit, University of Cape Town, Cape Town, South Africa,; 35. Department of Psychiatry, University of British Columbia, Vancouver, Canada; 36. Department of Psychiatry, Seoul National University College of Medicine, Seoul, Republic of Korea,; 37. Department of Neuropsychiatry, Seoul National University Hospital, Seoul, Republic of Korea,; 38. Institute of Human Behavioral Medicine, SNU-MRC, Seoul, Republic of Korea,; 39. Department of Psychiatry, Hanyang University College of Medicine, Seoul, Republic of Korea,; 40. Department of Psychiatry, Hanyang University Hospital, Seoul, Republic of Korea,; 41. Department of Child and Adolescent Psychiatry and Psychology, Hospital Clinic of Barcelona, Institut d’Investigacions Biomèdiques August Pi i Sunyer (IDIBAPS), Barcelona, Spain,; 42. Department of Medicine, University of Barcelona, Spain,; 43. SA MRC Unit on Risk and Resilience in Mental Disorders, Department of Psychiatry, Stellenbosch University, Stellenbosch, South Africa,; 44. Columbia University Medical College, Columbia University, New York, NY, USA; 45. Department of Radiology, Bellvitge University Hospital, Barcelona, Spain,; 46. Psychological Neuroscience Lab, CIPsi, School of Psychology, University of Minho, Braga, Portugal,; 47. Magnetic Resonance Image Core Facility, Institut d’Investigacions Biomèdiques August Pi i Sunyer (IDIBAPS), Barcelona, Spain,; 48. Department of Psychiatry, School of Clinical Sciences, Monash University, Melbourne, Australia,; 49. Monash Health, Melbourne, Australia,; 50. Division of Child and Adolescent Psychiatry, Jane & Terry Semel Institute For Neurosciences, University of California, Los Angeles, CA, USA,; 51. Departamento de Psicología Básica, Clínica y Psicobiología, Universitat Jaume I, Castellón de la Plana, Spain,; 52. Neuropsychiatry Laboratory, Department of Clinical Neuroscience and Neurorehabilitation, IRCCS Santa Lucia Foundation, Rome, Italy,; 53. Departments of Psychiatry, Neuroscience, Psychology, and Yale Child Study Center, Yale University, New Haven, CT, USA,; 54. Center for Brain and Mind Health, Yale University School of Medicine, New Haven, CT, USA,; 55. Graduate School of Systemic Neurosciences, Ludwig Maximilian University of Munich, Munich, Germany,; 56. ATR Brain Information Communication Research Laboratory Group, Kyoto, Japan,; 57. XNef, Inc.,; 58. Center of Mathematics, Computing and Cognition, Universidade Federal do ABC, Santo André, Brazil,; 59. Department of Integrative Medicine, National Institute of Mental Health And Neurosciences (NIMHANS), Bangalore, India,; 60. Department of Psychiatry, Columbia University Irving Medical Center, New York, NY, USA,; 61. Department of Social Psychology and Quantitative Psychology, Institute of Neurosciences, University of Barcelona, Barcelona, Spain,; 62. Neuroscience Institute, New York University School of Medicine, New York, NY, USA,; 63. Department of Psychiatry and Neuroscience, Icahn School of Medicine at Mount Sinai, NY, USA,; 64. Mental Illness Research, Education and Clinical Center (MIRECC), James J. Peters VA Medical Center, Bronx, NY, USA,; 65. Imaging Genetics Center, Mark and Mary Stevens Neuroimaging & Informatics Institute, Keck School of Medicine, University of Southern California, Los Angeles, CA, USA,; 66. Levvel, Amsterdam, the Netherlands,; 67. Department of Psychiatry, Arkin Mental Health Center, Amsterdam, the Netherlands,; 68. Mood, Anxiety, Psychosis, Stress and Sleep program, Amsterdam Neuroscience, Amsterdam, the Netherlands,; 69. Department of Anatomy & Neurosciences, Amsterdam University Medical Center, Vrije Universiteit Amsterdam, Amsterdam, the Netherlands,; 70. Department of Psychiatry, Amsterdam University Medical Center, Vrije Universiteit Amsterdam, Amsterdam, the Netherlands,; 71. Shanghai Mental Health Center, Shanghai Jiao Tong University School of Medicine, Shanghai, P. R. China,; 72. Cognitive Behavioral Therapy Center, Chiba University Hospital, Chiba, Japan,; 73. Department of Psychiatry, Seoul National University Hospital, Seoul, Republic of Korea,; 74. Yeongeon Student Support Center, Seoul National University College of Medicine, Seoul, Republic of Korea,; 75. SA MRC Unit on Risk and Resilience in Mental Disorders, Department of Psychiatry, Neuroscience Institute, University of Cape Town, Cape Town, South Africa,

## Abstract

Obsessive-compulsive disorder (OCD) is a disabling condition that is characterized by disruptions in distributed brain circuit dynamics. However, current network studies predominantly evaluate these circuits by measuring functional synchrony (connectivity) between pairs of regions of interest, potentially overlooking complex higher-order interactions. In this study, we applied a Hodge Laplacian topological framework to investigate these higher-order interactions in OCD. Using a large-scale resting-state fMRI dataset from the ENIGMA-OCD consortium (1,024 OCD patients and 1,028 healthy controls across 28 sites worldwide), we identified significant disruptions in topological loops spanning frontoparietal, default mode, and sensorimotor networks. Crucially, the edges constituting these abnormal loops largely lacked significant pairwise differences, highlighting higher-order multi-nodal disturbances. Subgroup analyses revealed that these disruptions were most pronounced in adult, medicated, and high-severity OCD patients. Our findings suggest that OCD pathology involves abnormal recurrent higher-order multi-region interactions, providing new insights into the brain’s functional organization and offering potential biomarkers for clinical application.

## Introduction

Obsessive-compulsive disorder (OCD) is a disabling psychiatric condition characterized by intrusive obsessions and repetitive compulsions that typically cause marked distress (1). These disturbing symptoms are related to a number of cognitive impairments in the disorder (2, 3). Given the complex nature of OCD pathology, it is believed that these symptoms arise from widespread abnormalities of multiple brain regions and networks, instead of the dysfunction of a single area (4). Neuroimaging studies of brain structure and function, together with studies of animal models, have revealed several potential pathological networks and circuits in OCD. One of the most well-studied models, involving the parallel cortico–striatal–thalamic–cortical (CSTC) circuits, contains recurrent feedback loops that are related to executive function, motor and response inhibition, working memory and reward processing (5, 6). More recently, involvement of other circuits like frontal-limbic and sensorimotor circuits has been recognized, reflecting both the potential heterogeneity and the systemic nature of the disorder (7, 8).

Functional magnetic resonance imaging (fMRI) as a powerful tool for investigating brain network function has provided evidence for these altered circuit connectivities in OCD (9). These brain network alterations involve widespread hypo-connectivity, specifically affecting connections inside or in-between the frontoparietal network (FPN), default mode network (DMN), salience network (SN), sensorimotor network (SMN), dorsal-attention network (DAN) and sub-cortical regions, although differences between OCD and HC are small (10–13). Conceptually, the repetitive nature of obsessions and compulsions may reflect a dysregulated signal flow trapped within these pathological feedback loops. However, conventional resting-state fMRI analyses have focused on pairwise functional connectivity (FC), assuming that network dysfunction can be decomposed and treating the brain as a collection of interactions between pairs of regions. These pairwise connectivities form the fundamental backbone structures of regional neural communication. However, a complex system such as the brain, where vast amounts of functionally segregated nodes are constantly interacting with one another, naturally contains higher-order integrations (18). Meanwhile, as neurobiological hypotheses of OCD (e.g., the CSTC circuit) are inherently defined by recurrent, multi-synaptic feedback loops, an edge-centric description may potentially neglect critical information about the multi-nodal topology of these pathological brain circuits. This motivates a complementary perspective that quantifies multi-nodal organization, especially loop-like structures formed by three or more regions, which can in principle vary even when individual pairwise FC changes might appear as statistically non-significant fluctuations (14–17). To systematically investigate these structures, we turn to topological data analysis (TDA).

TDA is an emerging mathematical framework grounded in algebraic topology and associated algorithms for probing the shape and structure of data (18). It is especially suitable for data that are intrinsically complex, multi-dimensional, multi-scalar and non-linear, such as molecular structure (19, 20), genomics (21), epidemiology (22) and medical imaging data (23, 24). It is particularly well-suited for neuroimaging data, as it copes with the complex and multi-dimensional nature of the data, while capturing global structural properties that are robust to noise and deformation (25). By modeling brain networks as simplicial complexes rather than simple graphs based on pairwise connectivities, TDA tracks features across dimensions via persistent homology (PH) (26), where 1-simplices represent pairwise interactions, 2-simplices represent three-node interactions (triangles), and 3-simplices represent four-node interactions (tetrahedra). It provides a principled way to analyze such higher-order organizations that cannot be reduced to local graph statistics (27, 28). Several attempts have been made to apply this PH framework to fMRI revealing novel biomarkers in various psychiatric disorders (25, 29–31). While PH is able to quantify the presence of abnormal loops (e.g., via Betti numbers), it cannot locate which specific brain regions or edges form these loops. This spatial ambiguity hinders the translation of topological findings into clinically interpretable results.

**Fig. 1.**
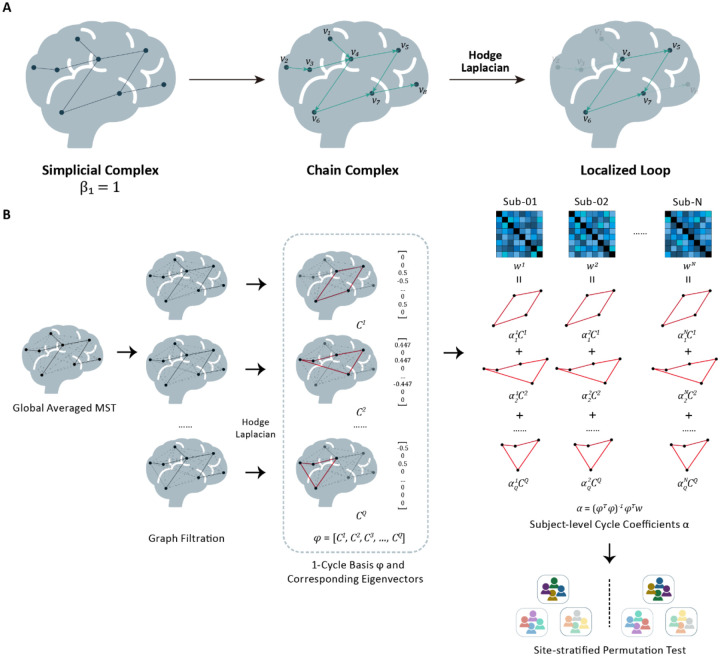
Overview of the Hodge Laplacian Topological Analysis. **A.** Conceptual Illustration. Traditional topological data analysis (left) can detect whether a loop exists in a topological space (e.g., Betti number $\beta_{1}=1$, indicating existence of 1 loop), but it does not identify *where* the loop is located or which edges contribute to it. The Hodge Laplacian framework uses the chain complex (middle), where edges are assigned with directions and organized into boundary operators. Decomposing edge flows using the Hodge Laplacian separates them into gradient, curl, and harmonic components. The harmonic component captures non-trivial cycles that are not boundaries of higher-dimensional structures. By isolating this component, we can localize the specific edges that form the loop (right), enabling spatial interpretation within the brain network. **B.** Computation of the Hodge Laplacian on resting state brain networks. First, we construct a group-level network skeleton using the maximum spanning tree (MST) to ensure connectivity while minimizing redundancy. From this skeleton, we derive a common 1-cycle basis representing independent loops in the group-averaged topology. Individual brain networks ware then projected onto the 1-cycle basis. The resulting cycle coefficients represent the expression of each loop in each subject. These subject-specific cycle coefficients are used as features for downstream statistical analysis (e.g., group comparisons or correlations with clinical measures in OCD).

Recently, Hodge Laplacian has been proposed to overcome these drawbacks. In contrast to conventional PH approaches, the Hodge Laplacian framework enables spatial localization of topological features. It has been applied to detect and locate cyclic structures, such as cell development trajectories (32), molecular biology (33), and, importantly, circuitry or loop structures in brain networks (34, 35). It assesses physiologically inherent higher-order structures - 1-cycles, or loops - and, crucially, localizes them with regard to well-established canonical brain networks. A 1-cycle encodes a closed, multi-edge route and can be viewed as a minimal substrate for recurrent interactions across distributed regions. From the perspective of a functional brain network, a 1-cycle encodes not only node-to-node information or signal transmission, but can be assumed to also take into account feedforward and feedback processes (36, 37). The Hodge Laplacian framework makes it possible to test disease effects directly on specific loop structures, rather than on single edges. This cycle-centric view is aligned with the broader idea that recurrent cyclic interactions form the foundation of brain dynamics regarding complex cognitive processes (38, 39), and therefore offers a mechanistically meaningful axis along which psychopathology may manifest (6, 40). An overview of the conventional TDA and Hodge Laplacian on a graph is presented in Figure 1A.

In this study, we applied the Hodge Laplacian-based TDA framework to a large-scale resting-state fMRI dataset from the ENIGMA-OCD working group. The method allows us to move beyond conventional pairwise connections to 1-cycles, which are closed topological loops formed by interactions of 1-simplices (i.e. FCs in a functional brain network) and represent the brain’s fundamental cyclic architecture. Crucially, we propose a shift in perspective regarding OCD pathophysiology: rather than viewing it as a collection of isolated abnormal pairwise connections, the repetitive and intrusive symptoms of OCD might reflect a disruption in the brain’s higher-order network structures, particularly within large-scale networks related to cognitive control and sensorimotor systems. Hence, by using this method we aim to provide new insights into the higher-order topological vulnerability of OCD brain networks.

## Results

### Hodge Laplacian reveals 1-cycle abnormalities in OCD regarding specific networks

To investigate higher-order topological abnormalities in OCD brain networks, we analyzed resting-state functional MRI (rs-fMRI) data from 2,052 participants (1,024 patients and 1,028 healthy controls) from the ENIGMA-OCD working group (see Supplementary Table S1 for sample sizes and information on scanning acquisition parameters of the included samples). We employed a Hodge Laplacian topological data analysis framework to construct a common 1-cycle basis derived from the group-averaged FC matrix. Subject-specific functional connectivity profiles were then projected onto this 1-cycle basis to obtain individualized 1-cycle coefficients. Using a Freedman-Lane permutation method on the coefficients for finding significantly discriminated 1-cycles between groups, we integrate a general linear model (GLM) within the site-stratified permutation test to control for age, sex, head motion, and scan site. The complete pipeline is presented in Figure 1B.

**Fig. 2.**
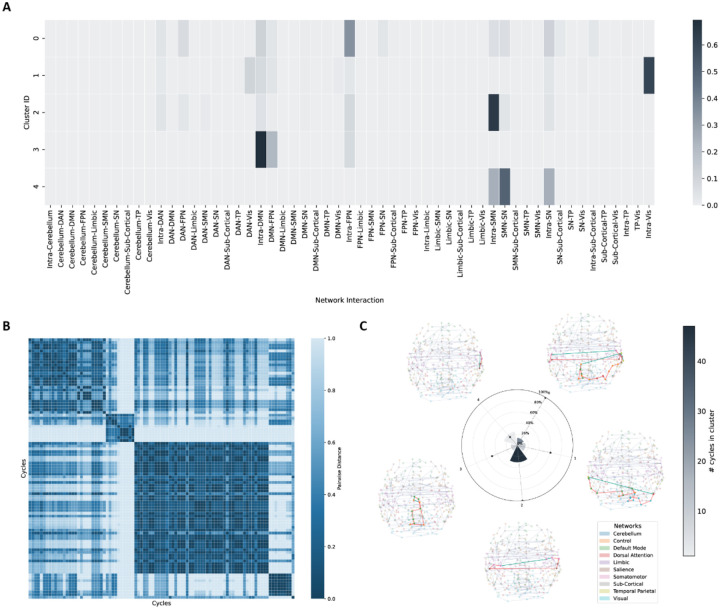
1-cycle abnormalities in main analysis. **A.** Agglomerative clustering results according to functional profiles of 1-cycles. **B.** Pairwise cosine distances between cycles. 5 distinct clusters are shown in the heatmap. **C.** Radial bar plot summarizing the following three cluster-level properties: Wedge height indicates the mean fraction of edges per 1-cycle that show significant edge-wise FC differences between OCD and controls, averaged across 1-cycles within each cluster. Bar color illustrates the number of significant 1-cycles in the cluster. The dot marks the cluster’s normalized mean 1-cycle length (mean number of edges per 1-cycle, normalized to the maximum across clusters). Visualizations of the most discriminating 1-cycles in each cluster are displayed around the plot. Global averaged maximum spanning tree structure is shown in the background. Different colors of nodes indicate different brain networks. Highlighted edges demonstrate the higher-order cycle structure, with green color indicating a significant difference in the functional connectivity strength of this edge between OCD and healthy controls. DAN: dorsal attention network, DMN: default mode network, FPN: frontal parietal network (labeled “Control” in the atlas), OCD: obsessive-compulsive disorder, SMN: somatomotor network, SN: salience network, TP: temporal parietal network, Vis: visual network.

We identified 93 significant discriminating 1-cycles in individuals with OCD compared to controls (family-wise error rate (FWER) corrected p<0.0001; Cohen’s d=0.223-0.283, partial ${\text{R}}^{2}=0.012-0.019$). The robustness and convergence of the permutation-based p-value estimation were confirmed by evaluating the null distribution and the stability of the p-value over 50,000 iterations (see Supplementary Figure S3 and S4). We summarized the functional profiles of these discriminating 1-cycles using an agglomerative clustering method. The result is illustrated in Figure 2A and 2B, where agglomerative clustering revealed 5 distinct functional profiles involving the FPN (labeled “Control” in the atlas), Visual (Vis)/DAN, SMN, DMN, and SMN/SN. Most of the 1-cycles belonged to the first (dominated by intra-FPN edges) and the third cluster (dominated by intra-SMN edges).

We summarized cluster-level properties in a radial bar plot (Fig. 2C) and visualized the most discriminating 1-cycle from each cluster around the plot. Critically, these topological alterations were largely independent of pairwise connectivity differences. The majority of edges constituting these abnormal cycles did not exhibit significant group differences in standard FC analysis (edges with green color), indicating that Hodge-Laplacian-derived 1-cycles captured higher-order, multi-nodal disruptions that are not detectable when assessing pairwise connectivity changes. We further investigated the connectivity strengths of these 1-cycles by comparing the averaged FC of every edge between OCD and HC participants. Specifically, individuals with OCD exhibited weaker FCs in these edges, indicating weaker integration in these network-specific cycles that mostly spanned both hemispheres.

### Similar yet different functional profiles for 1-cycles in different subgroups

To discover whether the observed topological alterations were related to specific clinical features, we applied the same Hodge Laplacian and permutation framework to patient subgroups stratified by age, medication status, symptom severity, and age of onset. Table 1 summarizes the Hodge Laplacian results for each subgroup. All subgroups were compared against HC. All subgroups showed significant between group differences on 1-cycles except the pediatric sample.

**Fig. 3.**
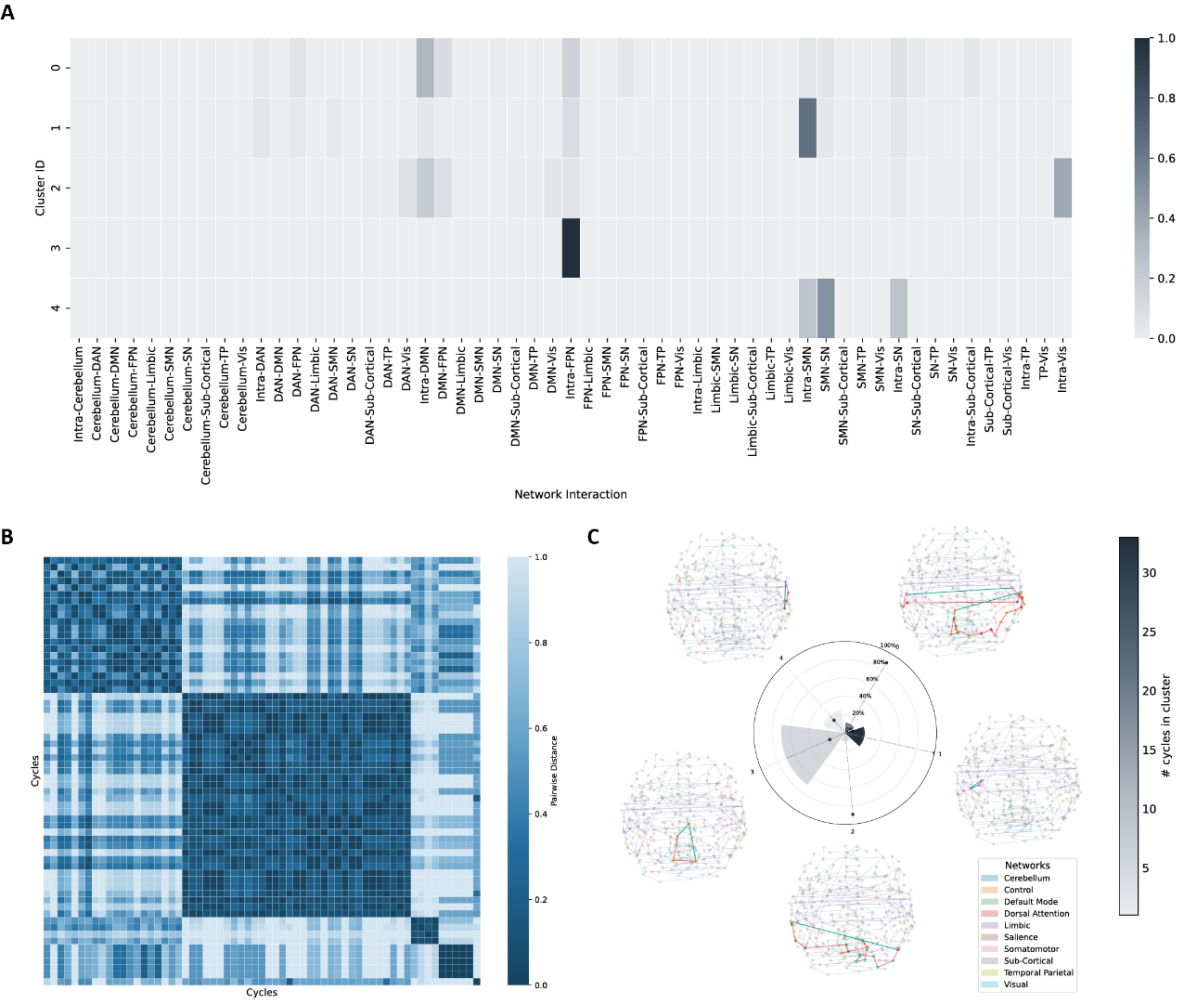
1-cycle abnormalities in adult sample. **A.** Agglomerative clustering results for adult samples according to functional profiles of 1-cycles. **B.** Pairwise cosine distances between cycles. 5 distinct clusters are shown in the heatmap. **C.** Radial bar plot summarizing the following three cluster-level properties: Wedge height indicates the mean fraction of edges per 1-cycle that show significant edge-wise FC differences between OCD and controls, averaged across 1-cycles within each cluster. Bar color illustrates the number of significant 1-cycles in the cluster. The dot marks the cluster’s normalized mean 1-cycle length. Visualizations of the most discriminating 1-cycles in each cluster are shown around the plot. DAN: dorsal attention network, DMN: default mode network, FPN: frontal parietal network (labeled “Control” in the atlas), OCD: obsessive-compulsive disorder, SMN: somatomotor network, SN: salience network, TP: temporal parietal network, Vis: visual network.

Among the subgroups that showed significant results, adult, medicated and high-severity subgroups exhibited the highest significance. For the adult sample (903 OCD patients and 914 matching HC participants), 63 discriminating 1-cycles were found (FWER corrected p=0.0001; Cohen’s d=0.237-0.304, partial ${\text{R}}^{2}=0.013-0.021$). The functional profiles (Figure 3) were similar to the main analysis, however, the adult sample showed more discriminating cycles in the first (intra-DMN edges) and second (intra-SMN edges) cluster.

**Fig. 4.**
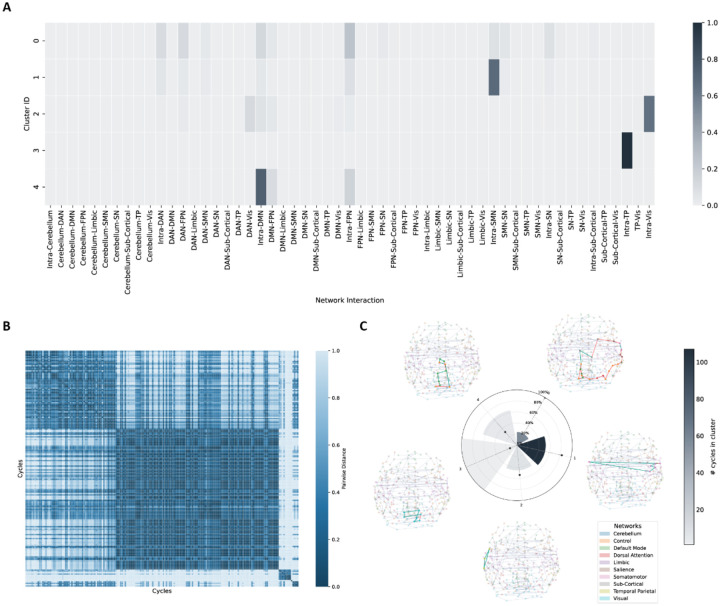
1-cycle abnormalities in medicated sample. **A.** Agglomerative clustering results for medicated samples according to functional profiles of 1-cycles. **B.** Pairwise cosine distances between cycles. 5 distinct clusters are shown in the heatmap. **C.** Radial bar plot summarizing the following three cluster-level properties: Wedge height indicates the mean fraction of edges per 1-cycle that show significant edge-wise FC differences between OCD and controls, averaged across 1-cycles within each cluster. Bar color illustrates the number of significant 1-cycles in the cluster. The dot marks the cluster’s normalized mean 1-cycle length. Visualizations of the most discriminating 1-cycles in each cluster are shown around the plot. DAN: dorsal attention network, DMN: default mode network, FPN: frontal parietal network (labeled “Control” in the atlas), OCD: obsessive-compulsive disorder, SMN: somatomotor network, SN: salience network, TP: temporal parietal network, Vis: visual network.

In the medicated sample (456 OCD patients and 683 matching HC participants), 179 discriminating 1-cycles were found (FWER corrected p=0.0001; Cohen’s d=0.307-0.474, partial ${\text{R}}^{2}=0.020-0.047$) which yielded the most significant result among the analyses. The medicated subgroup also showed disruptions, mostly in intra-FPN and intra-SMN connections, consistent with the main analysis (Figure 4). For the unmedicated sample (356 OCD patients and 420 matching HC participants), only one discriminating 1-cycle was identified (FWER corrected p=0.0139; Cohen’s d=0.381, partial ${\text{R}}^{2}=0.034$). This 1-cycle reflected a rather complex functional profile including several different networks (see Supplementary Figure S5).

**Fig. 5.**
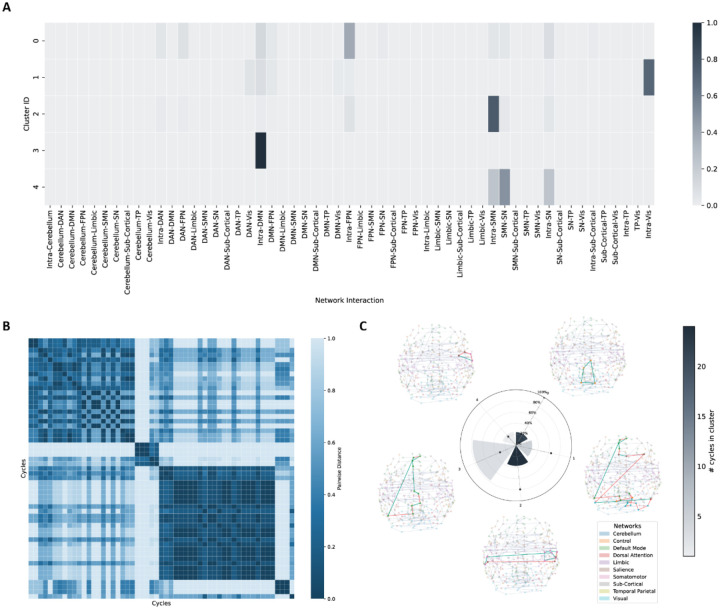
1-cycle abnormalities in high-severity sample. **A.** Agglomerative clustering results for high-severity samples according to functional profiles of 1-cycles. **B.** Pairwise cosine distances between cycles. 5 distinct clusters are shown in the heatmap. **C.** Radial bar plot summarizing the following three cluster-level properties: Wedge height indicates the mean fraction of edges per 1-cycle that show significant edge-wise FC differences between OCD and controls, averaged across 1-cycles within each cluster. Bar color illustrates the number of significant 1-cycles in the cluster. The dot marks the cluster’s normalized mean 1-cycle length. Visualizations of the most discriminating 1-cycles in each cluster are shown around the plot. DAN: dorsal attention network, DMN: default mode network, FPN: frontal parietal network (labeled “Control” in the atlas), OCD: obsessive-compulsive disorder, SMN: somatomotor network, SN: salience network, TP: temporal parietal network, Vis: visual network.

In the high-severity subgroup (414 OCD patients with Y-BOCS＞25 and 762 matching HC participants), 55 discriminating 1-cycles were found (FWER corrected p=0.0001; Cohen’s d=0.306-0.410, partial ${\text{R}}^{2}=0.020-0.036$). Most of the discriminating 1-cycles were clustered in the first (intra-FPN) and third (intra-SMN) cluster, also corresponding to the main analysis (Figure 5). In the low-severity subgroup (501 OCD patients with Y-BOCS≤25 and 880 matching HC participants), only 6 discriminating 1-cycles were found (FWER corrected p=0.0056; Cohen’s d=0.285-0.309, partial ${\text{R}}^{2}=0.017-0.021$). Half of the 1-cycles were related to intra-SMN connections (see Supplementary Figure S6).

We also performed the subgroup analysis on groups with different ages of onset of the disorder. Adult-onset OCD patients (388 OCD patients with age of onset ≥18 and 663 matching HC participants) showed 12 discriminating 1-cycles (FWER corrected p=0.0005; Cohen’s d=0.334-0.390, partial ${\text{R}}^{2}=0.023-0.031$). Half of them were assigned to the first cluster, mainly consisting of intra-SMN edges (see Supplementary Figure S7). Interestingly, none of the 1-cycles have intra-Vis connections, which differentiated this subgroup from the main analysis and the previous subgroups. The early-onset samples (305 OCD patients with age of onset ＜18 and 567 matching HC participants) showed 5 discriminating 1-cycles (FWER corrected p=0.01; Cohen’s d=0.369-0.387, partial ${\text{R}}^{2}=0.028-0.031$). However, it was hard to find a shared pattern among these 1-cycles, most of them had complex interactions of different networks (see Supplementary Figure S8).

### Sensitivity analysis

To verify the robustness of our method against spurious topological differences, we performed a split-half reliability analysis by repeating the full Hodge Laplacian workflow on two randomly partitioned subgroups of healthy controls. We did not find any differences regarding the 1-cycle structures between the two randomly split HC participants (p=0.429).

Furthermore, to confirm that the significant 1-cycles reflect higher-order loop properties without being biased by the underlying tree structure, we decomposed the 1-cycle coefficients into $\alpha_{\text{MST}}$ (tree) and $\alpha_{\text{Extra}}$ (off-tree) edge contributions by separately projecting these two parts on the 1-cycle basis. All 93 discriminating 1-cycles in the main analysis showed significant group differences on $\alpha_{\text{Extra}}$, but only 41 of them had different $\alpha_{\text{MST}}$. This indicated that the MST structures did not necessarily affect the significance (see Supplementary Table S2). On the contrary, most of the cycles were significant only after the non-MST edges were added.

## Discussion

Using a Hodge-Laplacian-based TDA framework, this study provides first evidence for an abnormal higher-order network topology in OCD. By localizing abnormal 1-cycles in a multi-site resting-state mega-analysis, results showed distinct groups of altered cycles in OCD spanning FPN, Vis/DAN, SMN, DMN, and SMN/SN networks. Similar patterns of higher-order topological disruptions were confirmed in adult, medicated, and high-severity subgroups. By comparing the results to conventional FC analyses based on pairwise connectivity, we found that these discriminating 1-cycles were largely independent from conventional FC. Together, these findings extend current models of OCD pathology by highlighting disruptions in multi-nodal feedback structures, in addition to isolated pairwise connections.

Our analysis revealed that the most discriminating 1-cycles were primarily composed of edges without significant between-group differences in edge-wise linear mixed effects (LME) analysis, indicating that OCD-related abnormalities are not only reflected in individual connections but also in the manner in which edges cohere into recurrent multi-node routes. By elevating inference from edges to loops, our results suggest that OCD pathophysiology may involve a disruption in higher-order coordination among large-scale systems. The abundance of discriminating 1-cycle effects in our analysis supports the long-standing view that OCD is fundamentally a disorder with distributed abnormal circuit dysfunction (7, 41, 42), while adding a new, localizable descriptor of recurrent cyclic structures that is largely invisible to standard FC analyses.

The disrupted 1-cycles were identified predominantly in the FPN, DMN, SMN, and Vis, which are known to be closely related to impaired cognitive control and sensorimotor function in the disorder, as suggested by prior resting state studies (9, 43–46). FPN-DMN interactions support the flexible alternation between externally oriented executive control and internally generated thoughts, with these abnormalities potentially causing impaired control over internally generated signals (10, 13, 47). SMN and Vis regions, moreover, are related to the generation and monitoring of motor behavior and sensory information (7, 48). Clinical studies indicated that altered motor control and sensory phenomena (SP) are experienced by a large proportion of OCD patients, which contributed to several domains of repetitive compulsive behaviors like ordering, counting or arranging (43, 49).

Our results therefore extend the current understanding of OCD pathophysiology by indicating higher-order loop abnormalities in networks involved in these cognitive processes and functions, even when individual edges show only subtle changes. There is good reason to assume that alterations in these processes contribute to the core symptoms of OCD when regarding thoughts and behavior. We hypothesize that these higher-order networks may rely more heavily on recurrent, closed-loop integration to maintain stability, such that abnormal re-entrant interactions within these networks might lead to cognitive malfunction and specific OCD symptomatology. As a concrete example, impaired cognitive control as a consequence of abnormal higher-order FPN function may cause obsessive thoughts to be insufficiently down-regulated and to be repeatedly reprocessed or to persist (50, 51). In parallel, sensory evidence, attentional sampling, and action execution may be continuously re-entered but rarely resolved due to a deficit in evaluating “completion”(49, 50, 52). Although edge-wise changes have also been observed when analyzing these cognitive impairments in OCD, we suggest that these dysfunctions are actually hierarchical. Notably, while classical OCD models prominently feature subcortical structures, our 1-cycle findings were predominantly cortical. This discrepancy suggests that subcortical dysfunctions might manifest more fundamentally as 0-dimensional, edge-level pairwise alterations. It is tempting to speculate that loop-level topology may reflect a global vulnerability in recurrent integration, whereas edge-wise differences may reflect more localized disruptions in point-to-point neural communication.

In addition, subgroup analyses suggest an additional, clinically meaningful implication of the 1-cycle abnormalities: the 1-cycle effects were strongest in adult, medicated and high-severity patients, whereas pediatric, unmedicated and low-severity subgroups showed little-to-no detectable effects. This mirrors prior ENIGMA-OCD resting-state findings at the edge level, where ROI-to-ROI differences were most evident in adult, medicated and high-severity subgroups, while pediatric, unmedicated and low-severity subgroups showed no significant group differences despite comparable directionality in some effect sizes (13). Such clinical burden related effects also appeared in white matter and brain volume abnormalities in OCD patients (53–55). We hypothesize that both pairwise FC alterations and Hodge 1-cycle abnormalities may reflect a more trait-like re-organization of the brain that becomes prominent with greater cumulative symptom burden, prolonged illness course, and/or treatment exposure.

Several limitations should be considered. First, given the cross-sectional nature of the ENIGMA dataset, we cannot determine the direction of causality. As in previous ENIGMA OCD analyses, we found significant topological disruptions especially in medicated and adult subgroups. It is difficult to distinguish whether stronger 1-cycle abnormalities reflect treatment exposure, greater severity, longer duration, or a trait marker for OCD pathology. This remains to be tested in future studies. Second, to ensure statistical comparability across thousands of subjects, we projected individual data onto a group-level 1-cycle basis derived from the group-averaged network. While this approach robustly identifies shared pathological backbones, it potentially neglects some individualized topological features, while also restraining the interpretation inside this specific dataset. Third, our analysis was performed on samples from existing studies worldwide, using different scanners and without harmonized data collection protocols. Although we have used a site-stratified permutation scheme together with correction of site-effects in the GLM model to minimize the heterogeneity of the multi-site data, the presence of additional (e.g. scanner related) site effects cannot fully be excluded. Finally, we restricted our analysis to 1-dimensional harmonic cycles to specifically target the feedback loop hypothesis; exploring higher-dimensional features (e.g., 2-dimensional voids) may reveal even more complex organizational alterations in OCD. However, it should be noted that there is the assumption that topological structures exceeding the 2-dimension do most probably not exist in the human brain (56). Considering TDA is still an emerging area, especially in neuroimaging, further research is required to develop a better understanding of the physiological substrates of human brain topology.

In conclusion, with the help of the Hodge Laplacian framework, we were able to unveil an up-to-now uninvestigated topological dimension of OCD pathology. We identified a set of disrupted, weakened higher-order functional loops, spanning frontoparietal, default mode, sensorimotor, and visual networks in patients. Crucially, these higher-order loop abnormalities are largely independent of pairwise connectivity strengths and suggest a circuit-based brain architecture in which pathology manifests not only as global pairwise dysconnectivity, but as a hierarchical disruption of distributed recurrent integration. By moving beyond the ‘edge-centric’ view to a ‘cycle-centric’ perspective, our framework provides a mathematically rigorous tool to map the disrupted circuitries that might underlie the repetitive and intrusive nature of OCD symptomatology. As many psychiatric disorders are increasingly understood as disorders of distributed circuit dynamics rather than isolated regional lesions (57–59), we believe the Hodge Laplacian framework has a great potential to probe the still largely unexplored higher-order topology of psychopathology.

## Materials and Methods

### Study samples

We analyzed data provided by the ENIGMA-OCD working group (https://enigma.ini.usc.edu/ongoing/enigma-ocd-working-group/). 1,024 OCD patients and 1,028 healthy controls from 28 sites, in total 2,052 subjects, were finally included in the study (see Supplementary Methods S1 for detailed inclusion/exclusion criteria). The diagnosis of OCD was based on DSM-IV(-TR) or DSM-5 criteria, the Yale-Brown Obsessive Compulsive Scale (Y-BOCS) and the Children’s Y-BOCS (CY-BOCS) were used for assessing symptom severity. All healthy participants were free of past and present diagnosis of psychiatric disorders as well as free of any psychotropic medication use at the time of inclusion. All studies were approved by the local institutional review board and participants provided written informed consent.

### Image acquisition and processing

Structural T1-weighted (T1w) and resting-state functional MRI data were acquired at 1.5 or 3 tesla and preprocessed locally at each site. rs-fMRI data were obtained for 4–12 min with a repetition time ranging between 700 and 3500 ms (see supplementary table). The images were analyzed using the fMRIPrep-based Harmonized AnaLysis of Functional MRI pipeline (HALFpipe, versions 1.0.0 to 1.2.1) (60), following standardized ENIGMA protocols for functional imaging (see http://enigma.ini.usc.edu/protocols/functional-protocols/).

Preprocessing included motion correction, slice timing and susceptibility distortion correction (if available), and spatial normalization. Denoising was performed after resampling the images to standard space and included spatial smoothing and grand mean scaling. ICA-AROMA and anatomical component correction (aCompCor) were used to regress out motion artifacts related to head motion, white matter (WM), and cerebrospinal fluid (CSF). Images were smoothed with a 6 mm full-width at half-maximum kernel.

Original ROIs selection included 400 cortical regions from the Schaefer-400 atlas with 17 networks, 17 sub-cortical regions from Harvard–Oxford atlas, and 17 cerebellar regions from Buckner-17 atlas. After excluding ROIs with less than 80% voxel coverage in participants, which may have resulted in the omission of some key regions in OCD pathology, four 6-mm spherical ROIs of amygdala and accumbens (ventral striatum) were added into the analysis based on the peak coordinates from NeuroSynth. Time series were then extracted from these 318 regions. A Gaussian-weighted high-pass filter with the width of 125 s was applied to the time series.

### Spectral graph theory

A FC matrix of brain network can be represented as a weighted undirected complete graph G=(V,E,w), where the vertex set V corresponds to ROIs, edge set E encodes FCs and w represents edge weights. In spectral graph theory, the graph G is represented by its adjacency matrix and Laplacian matrix. The adjacency matrix A contains the functional coupling between ROIs, and can be expressed as:

#(1) $$A(i,j)=\left\{\begin{array}{l} w_{ij},\left(v_{i},v_{j}\right)\in E\\ 0,\left(v_{i},v_{j}\right)\notin E \end{array}\right.$$

The Laplacian matrix is defined as $L_{0}=D-A$, where D is the degree matrix of G with its diagonal elements being the degrees of nodes (61). Specifically, the $L_{0}$ matrix can be expressed as:

#(2) $$L_{0}(i,j)=\left\{\begin{array}{c} \text{deg}\left(v_{i}\right),i=j\\ -1,i\neq j\;\text{and}\;\left(v_{i},v_{j}\right)\in E\\ 0,i\neq j\;\text{and}\;\left(v_{i},v_{j}\right)\notin E \end{array}\right.$$

$L_{0}$ is considered as a central operator in spectral graph theory. Its spectrum captures the fundamental features of the network’s structure, including connectedness and the presence of modular organization. Importantly, the number (multiplicity) of zero eigenvalues of $L_{0}$ corresponds to the number of connected components of the graph G. The proposed Hodge Laplacian generalizes $L_{0}$ from graphs to simplicial complexes, enabling spectral analysis of higher-dimensional topological structures such as cycles.

### Simplicial complex and chain complex

While the graph representation G=(V,E,w) captures pairwise functional couplings, many organizational features of brain networks arise from higher-order multi-nodal interactions that cannot be described solely by edges. This can be achieved by generalizing the graph into the simplicial complex as its higher-order representation. For the k-th dimension, a k-simplex $\sigma^{k}=\left\{v_{0},v_{1},\ldots ,v_{k}\right\}$ is a finite set of k+1 distinct vertices. Geometrically, for instance, a 0-simplex is a vertex which will be a point/node, 1-simplex is an edge with 2 vertices, and a 2-simplex is a triangle with 3 vertices. A simplicial complex K is then a collection of simplices up to a certain dimension.

In order to calculate the simplicial complex, one needs to define the orientation of a simplex that belongs to any above-0 dimension. Defining an orientation is necessary as signed edges are required for linear algebra operations to be applied. The orientation is usually specified according to the ordering of the vertices, either ascending or descending. For example, a 1-simplex $\sigma^{1}=\left\{v_{0},v_{1}\right\}$ is an edge and can be denoted as either $v_{0}-v_{1}$ or $v_{1}-v_{0}$, which means its direction is from $v_{1}$ to $v_{0}$ or from $v_{0}$ to $v_{1}$. With oriented k-simplices, we can sum them up and form a k-th chain group $C_{k}$. In other words, once every simplex has a direction (orientation), we can treat them as if they are signed building blocks and take linear combinations of them. All such combinations together form a vector space called the k-th chain group, denoted $C_{k}$. It is important to note that these assigned directions only represent higher-order statistical dependencies, rather than direct, causal neuronal transmission in the context of functional brain networks.

A chain complex is then defined as a sequence of these chain groups and the boundary operators are used for connecting chain groups from different dimensions (62). For an oriented k-simplex, the boundary operator $\partial_{k}:C_{k}\to C_{k-1}$ is defined as:

#(3) $$\partial_{k}\sigma^{k}=\sum_{i=0}^{k}(-1{)}^{i}\sigma_{i}^{k-1}=\sum_{i=0}^{k}(-1{)}^{i}\left[v_{0},v_{1},\ldots ,{\hat{v}}_{l},\ldots ,v_{k}\right]$$

where $\left[v_{0},v_{1},\ldots ,{\hat{v}}_{ı},\ldots ,v_{k}\right]$ is an oriented (k-1)-simplex generated from the vertices inside $\sigma^{k}$ except ${\hat{v}}_{ı}$. A simplex can be mapped to its boundaries by the boundary operator, for instance, an edge can be mapped to its two vertices, and a triangle to its three edges. This operation also guarantees that $\partial_{k-1}\partial_{k}=0$, indicating that the boundary of a boundary is empty. Thus, a chain complex can be expressed as:

#(4) $$\ldots \overset{\partial_{k+1}}{\to }C_{k}(K)\overset{\partial_{k}}{\to }C_{k-1}(K)\overset{\partial_{k-1}}{\to }\ldots \overset{\partial_{1}}{\to }C_{0}(K)\overset{\partial_{0}}{\to }0$$

Here, the boundary of the 0th chain group is an empty set. Also, for any n that exceeds the maximum dimension $k,C_{n}(K)$ is an empty vector space and the corresponding boundary operator is a zero map. See Supplementary Figure S1 for an illustration. Overall, in this way, we can organize simplices by dimension into vector spaces, and connect those spaces with boundary maps that encode how higher-dimensional structures attach to lower-dimensional ones.

From a geometric perspective, the topological feature of dimension k (which is usually referred to as k-dimensional hole) describes a closed structure that encloses a “void” and cannot be “filled in” or continuously contracted to a point within the space. For instance, a 1-dimensional hole manifests as a closed loop that is connected but surrounds an empty interior; it remains a distinct feature because it cannot be collapsed without altering the space’s topology. Algebraically, these features are formalized as homology groups, defined by the relationship between k-cycles and k-boundaries (see Supplementary Methods S2 for detailed mathematical definitions). In this study, as we only focus on the graph with the highest dimension of 1, a 0-dimensional cycle is then a connected component or a node, and a 1-dimensional cycle is a loop.

### Persistent homology and graph filtration

PH provides a framework for tracking how topological features—such as connected components, loops—emerge and disappear as a resolution or distance threshold varies. In classical PH, a sequence of nested simplicial complexes, which usually refers to a filtration, is constructed (63). The birth and death of k-dimensional features along this sequence are quantified using the duration of filtration values from birth to death known as persistence (64). The persistence is usually represented as one-dimensional intervals in a persistent barcode. Although the present study did not compute full persistence, the underlying concept of a filtration is essential for finding all possible 1-cycles in the graph.

For a weighted undirected complete graph G with m number of edges, the filtration process starts with the complete graph and is a sequence of sub-graphs ${\left(G_{\epsilon_{t}}\right)}_{t=1}^{m}$ of G:

#(5) $$G\subset G_{\epsilon_{m}}\subset \cdots \subset G_{\epsilon_{2}}\subset G_{\epsilon_{1}}$$

where $\epsilon_{1}<\epsilon_{2}<\cdots <\epsilon_{m}$ are the sorted edge weights from the graph (65, 66). Every subgraph is generated by removing one edge with the highest edge weights from the previous subgraph.

Graph filtration induces a natural birth–death decomposition. Birth edges forming a maximum spanning tree (MST) are responsible for creating connected components, while death (off-tree) edges are those excluded from the MST and are responsible for destroying loops (34). See Supplementary Methods S3 and Supplementary Figure S2 for an illustrative toy example. Importantly, adding any off-tree edge back to the MST creates a unique fundamental 1-cycle. This decomposition provides the basis for constructing the 1-cycle basis. With the graph filtration, we are able to analyze every possible 1-cycle structure, and also avoid an arbitrary connectivity threshold which is one of the major drawbacks of conventional graph analysis in neuroimaging (67, 68).

### Spectral simplicial complex and Hodge Laplacian

The graph Laplacian $L_{0}$, which applied on the classical graph theory analysis with nodal interactions, can be viewed as the 0-dimensional instance of a more generalized framework that extends spectral graph theory to simplices or higher-order interactions. This generalization is provided by the Hodge Laplacian, which is defined on the chain complex (34, 69). While the boundary operator $\partial_{k}$ defines the topological relationships algebraically, its implementation for data analysis requires a matrix form. For a chain complex C, its k-th boundary matrix $B_{k}$ is defined as:

#(6) $$B_{k}(i,j)=\left\{\begin{array}{c} 1,\text{if}\;\sigma_{i}^{k-1}\subset \sigma_{j}^{k}\;\text{and}\;\sigma_{i}^{k-1}\sim \sigma_{j}^{k}\\ -1,\text{if}\;\sigma_{i}^{k-1}\subset \sigma_{j}^{k}\;\text{and}\;\sigma_{i}^{k-1}≁\sigma_{j}^{k}\\ 0,\text{if}\;\sigma_{i}^{k-1}⊄\sigma_{j}^{k} \end{array}\right.$$

Same as the boundary operator, the boundary matrices also satisfy that $B_{k}B_{k+1}=0$. The k-th Laplacian matrix can be expressed as:

#(7) $$L_{k}=B_{k}^{T}B_{k}+B_{k+1}B_{k+1}^{T}$$

There is no boundary for 0-dimensional simplices $\left(B_{0}=0\right)$, the 0-th Laplacian, which is the graph Laplacian $L_{0}=B_{1}B_{1}^{T}$. In this study, as the highest dimension of the graph is 1, the 2-nd boundary matrix on a graph-based simplicial complex is also zero. Thus the 1-st Laplacian is $L_{1}=B_{1}^{T}B_{1}$.

### Algebraic representation for 1-cycles and statistical analysis

In order to identify and perform statistical comparisons for k-cycles, we first need to perform the Hodge decomposition. A k-th chain group will be then decomposed into three orthogonal subspaces:

#(8) $$C_{k}(K)=img{\;B}_{k+1}⊕\text{ker}\;L_{k}⊕img\;B_{k}^{T}$$

The gradient component corresponds to chains induced by lower-dimensional potentials, the curl component consists of boundaries of higher-dimensional simplices, and the harmonic component comprises divergence-free and curl-free chains. The harmonic subspace provides a direct mathematical representation of the feedback cycles and recurrent information integration processes, which are isomorphic to the k-th homology group and non-trivial topological cycles (70). In this study, we mainly focus on the second subspace, which is $ker\;L_{k}$. Statistical analyses were performed on this harmonic component to isolate genuine k-cycle differences across subjects. First, we solve the eigen-decomposition of $L_{k}$:

#(9) $$L_{k}=U_{k}\Lambda_{k}U_{k}^{T}$$

where $U_{k}$ is the matrix of eigenvectors, and k is a diagonal matrix of eigenvalues. Here, as the generalization of spectral graph theory, the multiplicity of zero eigenvalues of $L_{k}$ is equal to the k-th Betti number $\beta_{k}$. Harmonic eigenvectors corresponding to the zero eigenvalues can be expressed in the basis of k-simplices. The numerical entries of the eigenvector are therefore the coefficients of these simplices in the corresponding simplest possible k-cycle. A non-zero coefficient indicates that the simplex contributes to the cycle, whereas a zero coefficient indicates non-participation. Thus, the pattern of non-zero entries in a harmonic eigenvector can help to localize which simplices form the underlying topological feature. Given a k-cycle ${𝒞}_{k}$ that belongs to the k-th chain group $C_{k}$, its algebraic representation can be expressed as:

#(10) $${𝒞}_{k}=\sum_{i=1}^{j}a_{i}\sigma_{i}^{k}$$

where $a_{i}\in R$ is the i-th simplex coefficient, $\sigma_{i}^{k}$ is the i-th oriented k-simplex, and the eigenvector of ${𝒞}_{k}$ has j number of entries.

In this study, we focus on the 1-cycles in the graph. Since the harmonic 1-subspace only has two constraints “cycle” $\left(img\;B_{2}=0\right)$ and “not a triangle boundary” $\left(img\;B_{1}^{T}=0\right)$, the solution that satisfies these constraints does not have to be unique. However, when a graph contains multiple cycles, the harmonic 1-cycle can assign non-zero weights to edges that are not part of the target loop, which makes the resulting 1-cycle representations spatially diffuse and difficult to interpret.

The graph filtration process splits the edges in a graph into two separated edge sets. The birth set contains the exact MST structure, which, by definition, contains no loops. Consequently, every edge in the network that is not part of the MST (the “off-tree” edges) completes a unique cycle when added back to the MST. This procedure generates a set of fundamental 1-cycles that are linearly independent and cover the entire cyclic topology of the brain network.

Since now every subgraph contains exactly one 1-cycle, we are able to obtain localized and interpretable loops without superfluous edges. We can then apply Hodge Laplacian to extract the only eigenvector in the subgraph that corresponds to the only zero eigenvalue in the $L_{1}$ matrix. Under this circumstance, all the non-zero entries of this eigenvector will be part of the 1-cycle. This eigenvector represents the harmonic flow (or circular current) that minimizes the energy along the loop. This provides a physically meaningful quantification of the cyclic pattern. The whole process will result in a 𝒬×m coefficient matrix for 1-cycle basis, containing eigenvectors with the size of 1×m for 𝒬 number of possible 1-cycles.

To ensure comparability across subjects, we used the global averaged graph as the network template, and create a common 1-cycle basis $\phi =\left[{𝒞}_{1},{𝒞}_{2},\ldots ,{𝒞}_{𝒬}\right]$. To quantify how strongly each subject expresses the canonical cycle basis, we projected subject-level functional network onto the common 1-cycle basis. Given a vectorized upper triangle entry of individual FC matrix w, we have:

#(11) $$w=\alpha_{1}{𝒞}_{1}+\alpha_{2}{𝒞}_{2}+\cdots +\alpha_{𝒬}{𝒞}_{𝒬}$$

where the 1-cycle coefficients $\alpha ={\left[\alpha_{1},\alpha_{2},\ldots ,\alpha_{𝒬}\right]}^{T}$ quantify the subject-level variances on edge-weight patterns expressed in the harmonic cycle basis. This allows us to effectively decouple higher-order circuit topology from simple pairwise connection strengths. We can estimate α using least-squares approach, as:

#(12) $$\alpha ={\left(\phi^{T}\phi \right)}^{-1}\phi^{T}w$$

Then we can use α in discriminating between two groups of networks. The statistical significance is determined using the permutation test, which will be explained below.

### Characterization of OCD brain networks with the Hodge Laplacian

Here, we applied the Hodge Laplacian framework to the ENIGMA-OCD dataset to investigate these higher-order topological features in OCD brains. For each participant, we derived a 318 × 318 resting-state FC matrix based on the extracted time series using Pearson’s correlations with Fisher Z-transformation, vectorized the upper-triangular entries, and extracted a common 1-cycle basis from the group-averaged network. The basis consisted of 𝒬=50,086 harmonic 1-cycles, each represented as a vector in the edge space of dimension m=50403 (including all possible edges). Subject-level FC patterns were projected onto this common 1-cycle basis to obtain a cycle coefficient matrix of size 2,052 × 50,086 (subjects × cycles), following the procedure described in previous sections.

Because the dataset spans multiple international sites and a wide age range, covariate adjustment was necessary before assessing group differences in 1-cycle coefficients α. To achieve this, we employed a GLM. The model included the diagnostic group as the variable of interest, while controlling for age, sex, mean framewise displacement (FD), and scan site as nuisance covariates. To account for multi-site heterogeneity, site effects were modeled as fixed effects (using dummy variables with the first column removed to avoid multicollinearity). The fixed-effects model was chosen over random effects to avoid assumptions about the distribution of site-level variance. From this GLM, the group difference for each 1-cycle coefficient was quantified using the T-statistic. We favored this feature-level statistical adjustment over direct data harmonization (e.g., ComBat) because altering raw connectivity matrices could disrupt the rank-order of edge weights, thereby potentially distorting the true topological structures during the filtration process.

To incorporate the GLM model in the permutation framework, we used the Freedman-Lane permutation method (71). Briefly, we first fitted a reduced model containing only the nuisance covariates to the data. The resulting residuals were then permuted and added back to the predicted values from the reduced model to generate a null dataset. To preserve the inherent data structure of the multi-center study, the permutation of residuals was stratified by site (i.e., shuffling was restricted within each imaging center). This stratification ensures that the exchangeability assumption holds only among participants scanned under the same conditions, thereby accounting for potential site-related variance differences in the null distribution. This process was repeated 50,000 times, which was sufficient to ensure stable estimation of permutation-based p-values. Family-wise error rate (FWER) correction was implemented by comparing the T statistic of each cycle to the maximum T statistic observed across all cycles in each permutation. Effect sizes (Cohen’s d) were calculated based on the GLM parameters, and normalized by the residual standard deviation to account for covariates.

Significant 1-cycles (global FWER < 0.05) were further analyzed to identify their network-level functional profiles. Each FC edge was assigned to one of 55 functional interaction categories spanning 10 large brain networks defined by the atlases, including the intra- and inter-network connections. Each 1-cycle vector was binarized (edge present = 1 else 0) and transformed into a 55-dimensional functional profile by counting and normalizing edge participation across functional categories. Pairwise cosine distances between profiles were computed, and agglomerative clustering (average linkage, similarity threshold 0.5) was applied to identify cycle families sharing similar network-level organization. For visualization, we computed for each 1-cycle (i) its size as the number of constituent edges, and (ii) the fraction of constituent edges that were significant in edge-wise FC analysis (described in sensitivity analysis). Cluster-level values were obtained by averaging these metrics across 1-cycles within each cluster; mean length was normalized by the maximum cluster mean.

To further examine clinical heterogeneity and assess the influence of, e.g., medical treatment, OCD samples were further divided into subgroups, including patients with or without current use of psychotropic medication at time of scanning, with mild to moderate symptom severity (Y-BOCS≤25, low-severity) or moderate to severe (Y-BOCS＞25, high-severity), as well as adult patients with early (<18 years) or late (≥18 years) age of onset. Samples with <10 participants per group were excluded for each subgroup analysis. These subgroups correspond to subgroups in prior ENIGMA-OCD mega-analyses, and all the subgroups were compared against HC, using the same framework as in the main analysis.

### Sensitivity and Robustness Analyses

We performed several additional analyses to test the sensitivity and robustness of our method. First, to verify that the Hodge Laplacian framework does not yield spurious topological differences, we (site-stratified) partitioned the 1,028 HC participants into two matched subgroups and repeated the full Hodge Laplacian workflow—including 1-cycle extraction, projection, and Freedman–Lane testing. Theoretically, the two subgroups should not show any topology differences and the comparison should yield a non-significant result.

Second, as the crucial part of the Hodge Laplacian framework is the individual projection of the upper-triangular entries of FC, there is a certain probability that the significant 1-cycles strongly rely on the pairwise connections that already showed significant between group differences. To verify that the detected 1-cycles are actually higher-order abnormalities which are independent from the 0-dimensional connections, we used an LME model to find the between group differences on FC. Group was included as a fixed effect, age, sex and FD as covariates, and site ID as a random intercept, in line with the previous ENIGMA-OCD mega-analyses on the resting-state connectome(13). Multiple comparisons correction for the FC comparison was applied using Benjamini-Hochberg false discovery rate (FDR) correction. We also analyzed the FC differences in each subgroup. qFDR for each comparison was Bonferroni corrected (0.05/9 (for 9 contrasts) = 0.0055556) to account for the number of contrasts tested simultaneously. The significant FCs were then mapped onto the significant 1-cycles to visualize their overlapping properties, presented as green colored edges in the figures.

Third, considering that each 1-cycle is constructed by a large MST skeleton with only one additional non-MST edge, there is a possibility that the MST structure will strongly affect the significance of the 1-cycle and cause potential bias. To test this, we further decomposed the 1-cycle coefficient α into contributions along (i) edges belonging to the subject-specific MST and the (ii) remaining off-tree edges:

#(13) $$\alpha_{\text{MST}}={\left(\phi^{T}\phi \right)}^{-1}\phi^{T}w_{\text{MST}},\;\alpha_{\text{Extra}}={\left(\phi^{T}\phi \right)}^{-1}\phi^{T}w_{\text{Extra}},$$

where $w_{\text{Extra}}=w-w_{\text{MST}}$. This decomposition should reveal whether the MST edges (which always are the major part of the cycle) or the extra non-MST edge (which always closes the cycle) contribute more to the significance. We performed paired sample t-tests on these two subparts of α using the significant 1-cycles we got from the main analysis. FDR was used for multiple comparisons correction.

## Supplementary Material

Supplement 1

1

## Figures and Tables

**Table. 1.** Summary of permutation results regarding OCD vs HC across the main and subgroup analyses. Global p denotes the FWER-corrected permutation p value for the group effect. “Cycles (N)” indicates the number of significant 1-cycles. Effect sizes (Cohen’s d, partial ${\text{R}}^{2}$) are reported as ranges across significant 1-cycles.

	OCD (N)	HC (N)	Global p	Cohen’s d	Partial $R^{2}$	Cycles (N)
Pediatric	103	101	0.839	/	/	1
Adult	903	914	0.0001	0.237–0.304	0.013–0.021	63
Unmedicated	356	420	0.0139	0.381	0.034	1
Medicated	456	683	0.0001	0.307–0.474	0.020–0.047	179
Low-severity	501	880	0.0056	0.285–0.309	0.017–0.021	6
High-severity	414	762	0.0001	0.306–0.410	0.020–0.036	55
Early-onset	305	567	0.01	0.369–0.387	0.028–0.031	5
Adult-onset	388	663	0.0005	0.334–0.390	0.023–0.031	12
Main	1024	1028	0.0001	0.223–0.283	0.012–0.019	93

## Data Availability

The full ENIGMA OCD data are not publicly available in a repository as they might contain information that could compromise the privacy of research participants. Access to these data may be requested through the ENIGMA OCD Working Group data access procedures and is subject to approval and completion of the required data-use agreements. More information about the ENIGMA OCD Working Group can be found here: https://enigma.ini.usc.edu/ongoing/enigma-ocd-working-group/. The source codes for the Hodge Laplacian analysis, sensitivity analysis, visualization, as well as non–participant-level outputs in this study are openly available on Zenodo at https://doi.org/10.5281/zenodo.18861978. All other data needed to evaluate the conclusions are available in the Article and/or Supplementary Information.
