# Supplementary material for: Disrupted Higher-Order Topology in OCD Brain Networks Revealed by Hodge Laplacian – an ENIGMA Study
Source: bioRxiv. 2026 Mar 6:2026.03.04.709586. Preprint. [Version 1] doi: 10.64898/2026.03.04.709586 (PMC12991123; doi:10.64898/2026.03.04.709586)
Supplement: 1 [file NIHPP2026.03.04.709586V1-supplement-1.pdf]

bioRxiv preprint doi: https://doi.org/10.64898/2026.03.04.709586; this version posted March 6, 2026. The copyright holder for this preprint (which was not certified by peer review) is the author/funder, who has granted bioRxiv a license to display the preprint in perpetuity. It is made available under aCC-BY 4.0 International license.

Supplementary Text

Subject exclusion

The exclusion process was kept the same with the previous ENIGMA-OCD connectome research. Specifically, we excluded 2 healthy controls who were using psychotropic medication, 264 participants whose data failed neuroimaging quality control, 111 participants due to excessive motion, 315 participants with insufficient brain coverage and 151 participants from samples with <10 participants per group. Finally, 1024 OCD patients and 1028 healthy controls, in total 2052 subjects were included in the study.

Mathematical definitions of topological holes

k-dimensional hole describes a closed structure that encloses a “void” and cannot be “filled in” or continuously contracted to a point within the space. Mathematically, this geometric intuition is formalized as an algebraic construction: a structure that is closed but does not bound a higher-dimensional volume. For a chain complex C_k, we distinguish two fundamental subspaces. The collection of k-cycles Z_k is the kernel subspace, which is expressed as:

$$Z_k = ker\,\partial_k = \{\sigma^k \in C_k | \partial_k \sigma^k = 0\} \#(1)$$

Z_k contains all possible candidates of k-cycles, as long as they are a closed (the boundary is zero). The collection of k-boundaries B_k is the image subgroup, which is expressed as:

$$B_k = img\,\partial_{k+1} = \{\sigma^k \in C_k | \sigma^k = \partial_{k+1}\sigma^{k+1}, \sigma^{k+1} \in C_{k+1}\} \#(2)$$

This “closed-but-not-filled” condition implies that the cycle cannot be trivialized by the boundary operator. Specifically, a topological feature corresponds to a k-cycle (an element of $ker\,\partial_k$) that fails to be a k-boundary (an element of $img\,\partial_{k+1}$). Consequently, the k-homology class H_k is then represented as $H_k = Z_k/B_k$. Importantly, H_k is not a single cycle but an equivalence class of cycles. As long as the difference between two cycles is a boundary, they are considered as equivalent.

bioRxiv preprint doi: https://doi.org/10.64898/2026.03.04.709586; this version posted March 6, 2026. The copyright holder for this preprint (which was not certified by peer review) is the author/funder, who has granted bioRxiv a license to display the preprint in perpetuity. It is made available under aCC-BY 4.0 International license.

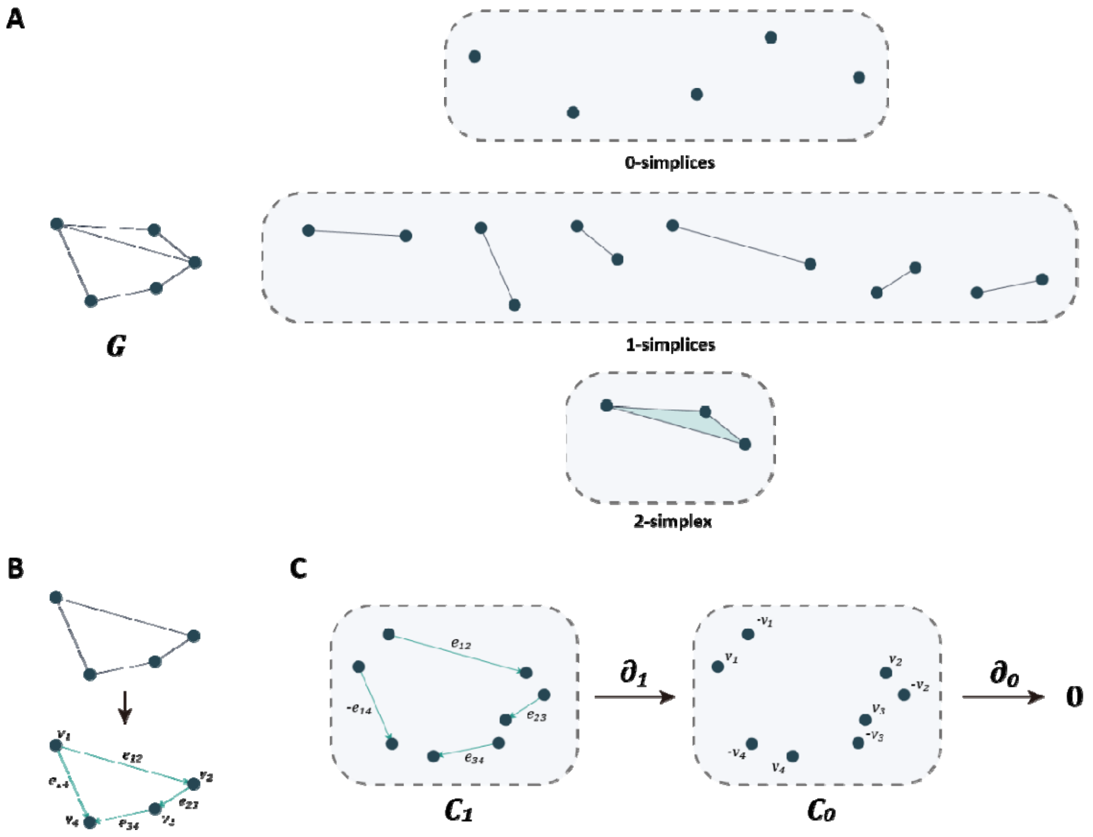

Fig. S1. Explanation of simplicial complex, chain complex and boundary operator. A. A toy graph and its associated simplices: 5 vertices (0-simplices), 6 edges (1-simplices), and 1 filled triangle representing a 2-simplex. **B.** For computation and the construction of chain complex, orientation is assigned to simplices to generate oriented chains. **C.** The resulting chain groups and and the action of the boundary operators (mapping oriented edges to signed endpoints) and (mapping 0-chains to the zero element).

bioRxiv preprint doi: https://doi.org/10.64898/2026.03.04.709586; this version posted March 6, 2026. The copyright holder for this preprint (which was not certified by peer review) is the author/funder, who has granted bioRxiv a license to display the preprint in perpetuity. It is made available under aCC-BY 4.0 International license.

Birth-death decomposition

Here we present a toy model of a 4-node graph as an example (Supplementary Figure S2). In the context of persistent homology, the evolution of topological features is typically quantified by Betti numbers, where β_0 represents the number of connected components and β_1 represents the number of cycles (loops), as demonstrated in the visualization. The red barcodes represent the 0-cycles or the connected components in the graph. Notice that once a component is born, it does not die, so all the connected components have ∞ death values which can be ignored. For a graph with n nodes, the total number $\mathcal{P}$ of birth values of connected components is $n-1$, which correspond to the β_0 number of the graph. The light red barcode which corresponds to the original complete graph is taken out because it doesn't have a birth value. The birth value set $\mathcal{B}(G)$ of the graph G is then an increasing set of edge weights. The green barcodes represent the 1-cycles or the loops in the graph. All the loops are naturally existing in the complete graph; thus, all the birth values are $-\infty$ and can be ignored. The death value set $\mathcal{D}(G)$ of the graph G is then a decreasing set of edge weights. The two sets together compose all edges in the graph since they cover all filtration values in the filtration process, thus we have:

$$m = \frac{n(n-1)}{2} = \mathcal{P} + \mathcal{Q} \#(3)$$

The total number $\mathcal{Q}$ of death values of loops is then $(n-1)(n-2)/2$, which correspond to the β_1 number of the graph.

bioRxiv preprint doi: https://doi.org/10.64898/2026.03.04.709586; this version posted March 6, 2026. The copyright holder for this preprint (which was not certified by peer review) is the author/funder, who has granted bioRxiv a license to display the preprint in perpetuity. It is made available under aCC-BY 4.0 International license.

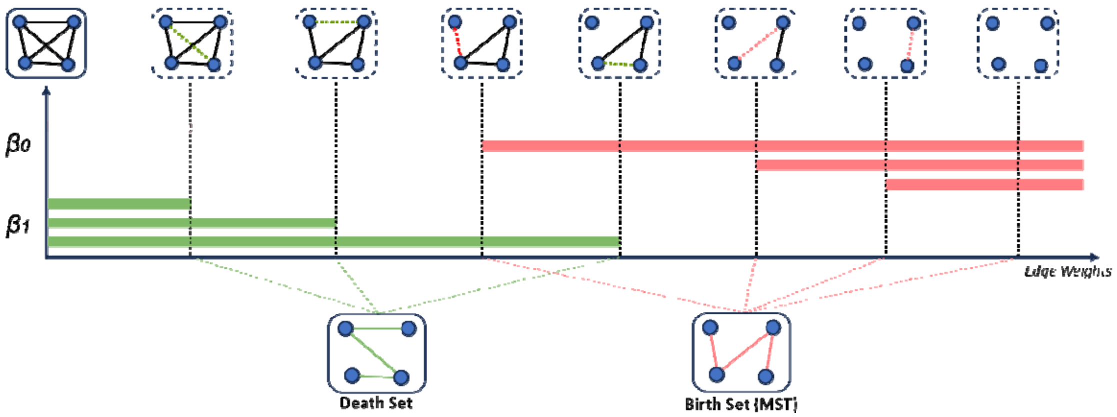

Fig. S2. Schematic of graph filtration process. Based on the concept of persistent homology, we perform the graph filtration according to sorted edge weights. The number of connected components ( ) and the number of cycles ( ) change at different filtration values. Notice that since a component has infinite death value and a cycle has infinite birth value, we can decompose edges into death set and birth set, and the birth set is exactly the maximum spanning tree (MST) structure of the graph.

bioRxiv preprint doi: https://doi.org/10.64898/2026.03.04.709586; this version posted March 6, 2026. The copyright holder for this preprint (which was not certified by peer review) is the author/funder, who has granted bioRxiv a license to display the preprint in perpetuity. It is made available under aCC-BY 4.0 International license.

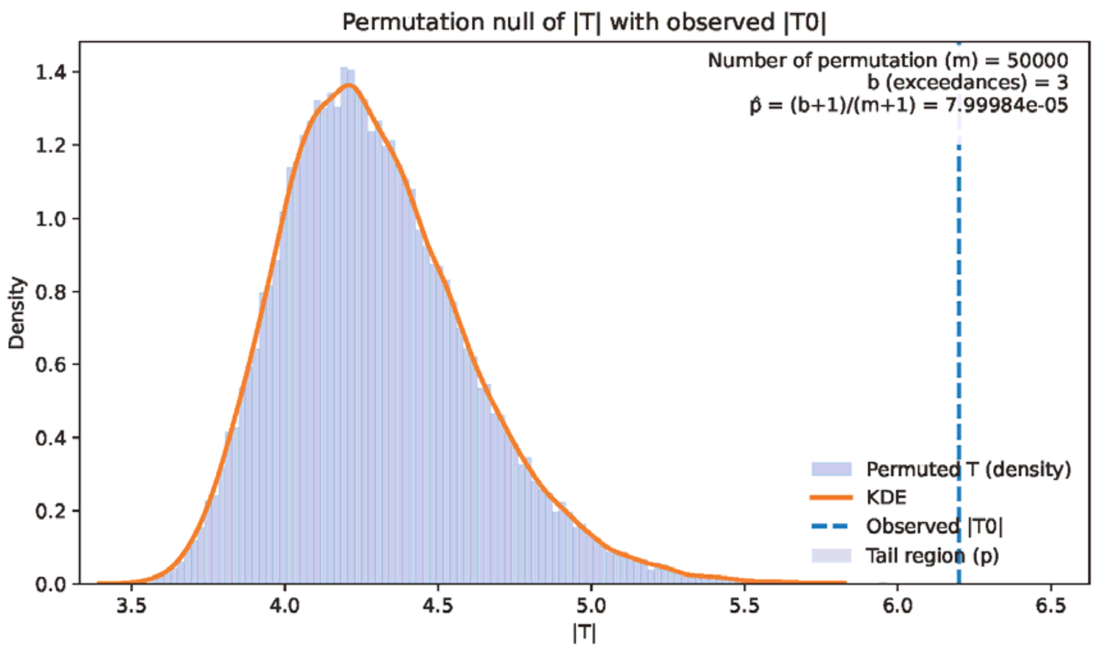

Fig. S3. Null distribution of the permutation test. The histogram and the kernel density estimation (KDE, orange curve) represent the empirical null distribution of the maximum absolute T-statistics obtained from 50,000 permutations from the main analysis. The vertical dashed line indicates the observed maximum absolute T-statistic ($|\mathbf{T0}|$). Only 3 out of 50,000 permutations exceeded the observed value, yielding a highly conservative $\mathbf{p}$-value of $\mathbf{p} \approx \mathbf{8} \times \mathbf{10^{-5}}$. This demonstrates that the significance of discriminating 1-cycles were highly unlikely to have arisen by chance, even after controlling for multiple comparisons.

bioRxiv preprint doi: https://doi.org/10.64898/2026.03.04.709586; this version posted March 6, 2026. The copyright holder for this preprint (which was not certified by peer review) is the author/funder, who has granted bioRxiv a license to display the preprint in perpetuity. It is made available under aCC-BY 4.0 International license.

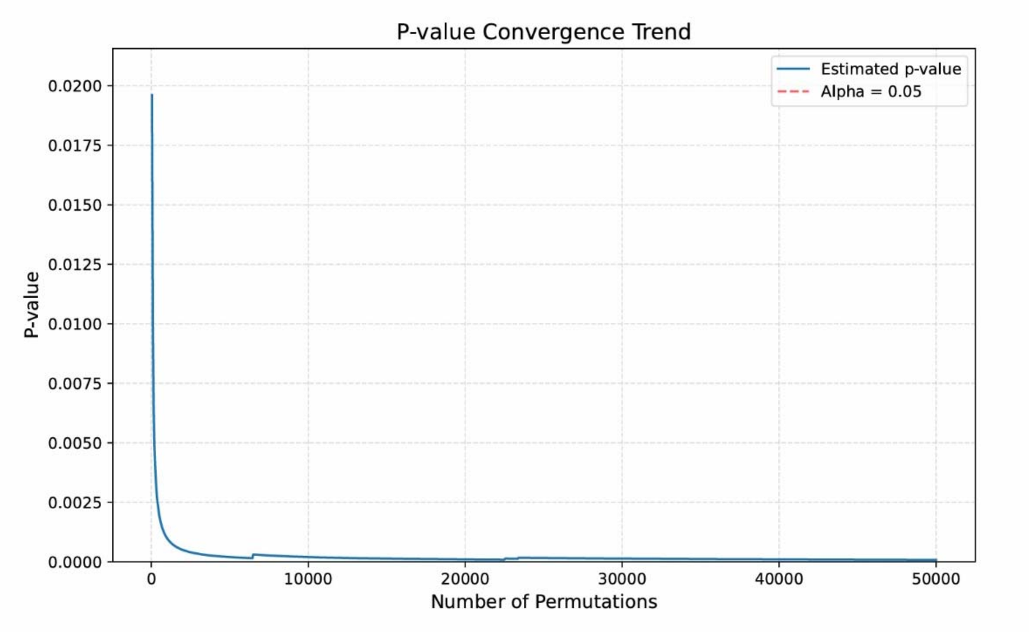

Fig. S4. Convergence of the estimated *p*-value relative to the number of permutations. The estimated *p*-value rapidly descended and stabilized below the significance threshold of 0.05. The observed convergence after approximately 10,000 iterations suggested that the permutation test has reached sufficient numerical precision to support the statistical significance of the results.

bioRxiv preprint doi: https://doi.org/10.64898/2026.03.04.709586; this version posted March 6, 2026. The copyright holder for this preprint (which was not certified by peer review) is the author/funder, who has granted bioRxiv a license to display the preprint in perpetuity. It is made available under aCC-BY 4.0 International license.

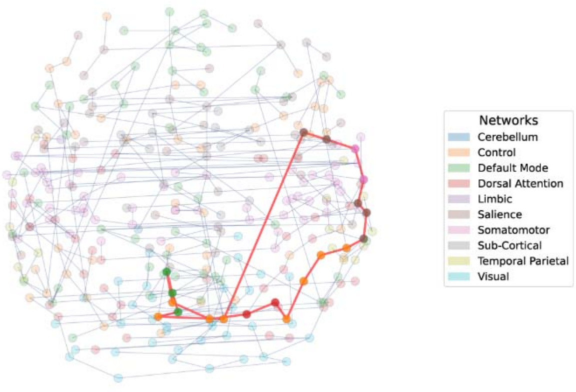

Fig. S5. Discriminating 1-cycle structure of unmedicated subgroup analysis. This 1-cycle showed interaction among default mode network, somatomotor network, dorsal attention network, salience network and control/frontal parietal network.

bioRxiv preprint doi: https://doi.org/10.64898/2026.03.04.709586; this version posted March 6, 2026. The copyright holder for this preprint (which was not certified by peer review) is the author/funder, who has granted bioRxiv a license to display the preprint in perpetuity. It is made available under aCC-BY 4.0 International license.

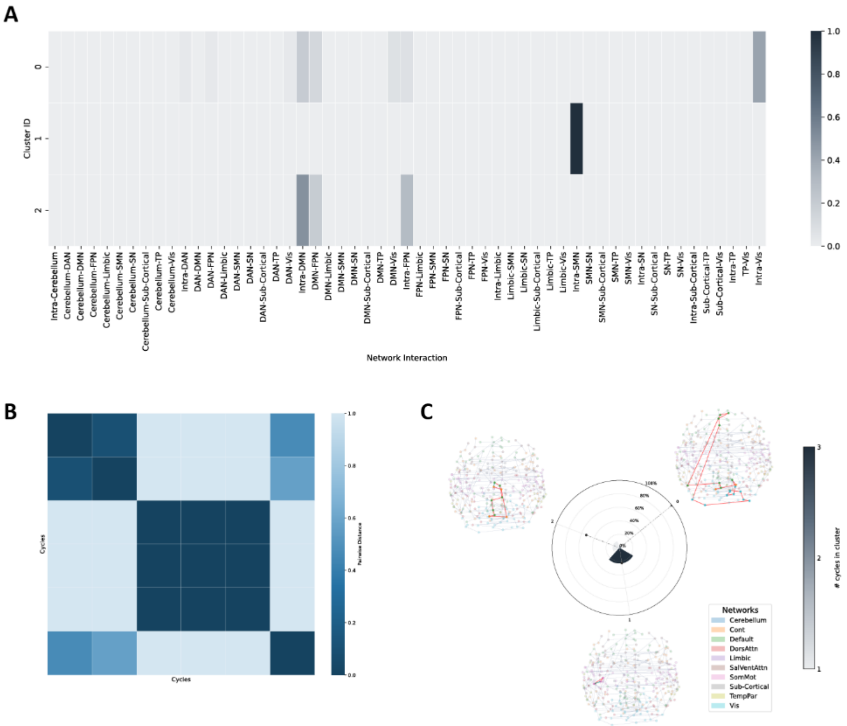

Fig. S6. 1-cycle abnormalities in low-severity sample. A. Agglomerative clustering results for low severity sample according to functional profiles of cycles. 3 clusters were found in this subgroup analysis, and half of them were related to intra-SMN connections. **B.** Pairwise cosine distances between cycles. **C.** Cluster-level results summarized by a radial bar chart, where each bar represents a cluster and its height indicates the proportion of significant edges. Bar color reflects the number of 1-cycles in the cluster, and a dot marker denotes the normalized mean 1-cycle size. Visualizations of the most discriminating 1-cycles in each cluster are shown around the plot. DAN: dorsal attention network, DMN: default mode network, FPN: frontal parietal network (labeled “Control” in the atlas), OCD: obsessive-compulsive disorder, SMN: somatomotor network, SN: salience network, TP: temporal parietal network, Vis: visual network.

bioRxiv preprint doi: https://doi.org/10.64898/2026.03.04.709586; this version posted March 6, 2026. The copyright holder for this preprint (which was not certified by peer review) is the author/funder, who has granted bioRxiv a license to display the preprint in perpetuity. It is made available under aCC-BY 4.0 International license.

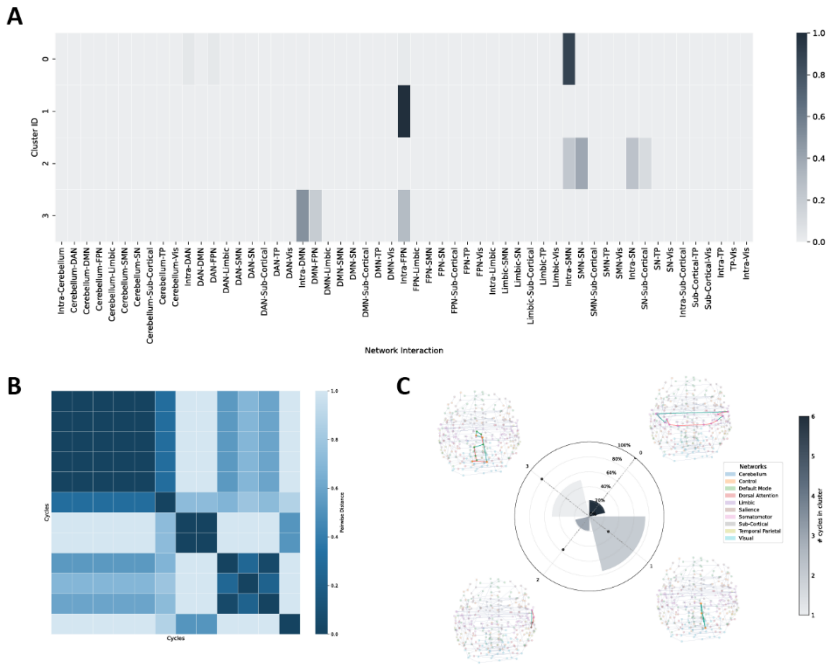

Fig. S7. 1-cycle abnormalities in adult-onset sample. A. Agglomerative clustering results for adult-onset sample according to functional profiles of cycles. 4 clusters were found in this subgroup analysis, and half of them were also related to intra-SMN connections. **B.** Pairwise cosine distances between cycles. **C.** Cluster-level results summarized by a radial bar chart, and visualizations of the most discriminating 1-cycles in each cluster are shown around the plot. DAN: dorsal attention network, DMN: default mode network, FPN: frontal parietal network (labeled “Control” in the atlas), OCD: obsessive-compulsive disorder, SMN: somatomotor network, SN: salience network, TP: temporal parietal network, Vis: visual network.

bioRxiv preprint doi: https://doi.org/10.64898/2026.03.04.709586; this version posted March 6, 2026. The copyright holder for this preprint (which was not certified by peer review) is the author/funder, who has granted bioRxiv a license to display the preprint in perpetuity. It is made available under aCC-BY 4.0 International license.

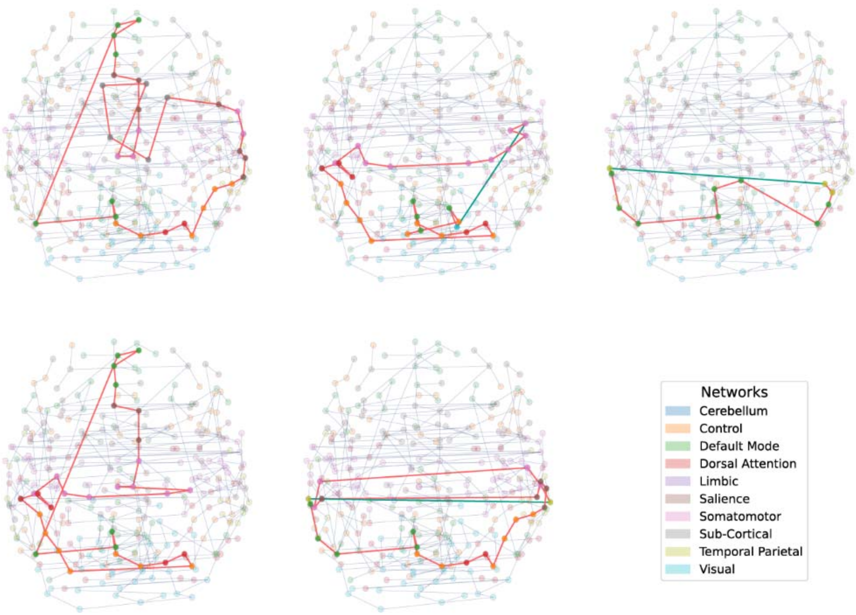

Figure S8. Visualization of 5 most discriminating 1-cycles in early-onset samples.

bioRxiv preprint doi: https://doi.org/10.64898/2026.03.04.709586; this version posted March 6, 2026. The copyright holder for this preprint (which was not certified by peer review) is the author/funder, who has granted bioRxiv a license to display the preprint in perpetuity. It is made available under aCC-BY 4.0 International license.

Table S1. Available information on sample sizes and scanning acquisition parameters used to obtain structural and functional resting-state data for included ENIGMA-OCD samples. TR=Repetition Time; TE=Echo Time.

bioRxiv preprint doi: https://doi.org/10.64898/2026.03.04.709586; this version posted March 6, 2026. The copyright holder for this preprint (which was not certified by peer review) is the author/funder, who has granted bioRxiv a license to display the preprint in perpetuity. It is made available under aCC-BY 4.0 International license.

bioRxiv preprint doi: https://doi.org/10.64898/2026.03.04.709586; this version posted March 6, 2026. The copyright holder for this preprint (which was not certified by peer review) is the author/funder, who has granted bioRxiv a license to display the preprint in perpetuity. It is made available under aCC-BY 4.0 International license.

Cycle ID	p_α_{MST}	p_α_{Extra}
**1821**	5.38E-03	1.94E-02
**1841**	N.S.	4.53E-04
**6650**	N.S.	2.11E-06
**7537**	N.S.	1.32E-03
**11046**	2.38E-06	7.31E-10
**16037**	2.57E-06	2.21E-08
**16885**	2.85E-08	9.14E-07
**21360**	1.02E-08	1.32E-07
**23403**	N.S.	2.01E-06
**24824**	2.29E-10	5.37E-05
**26488**	1.12E-05	3.30E-06
**29185**	6.26E-10	4.23E-06
**29619**	2.68E-10	1.23E-05
**31045**	N.S.	3.43E-06
**31972**	N.S.	6.71E-05
**32280**	1.49E-02	6.22E-04
**32684**	1.58E-02	3.40E-04
**36210**	N.S.	2.61E-07
**39647**	N.S.	1.23E-05
**40043**	N.S.	1.37E-05
**40817**	N.S.	7.31E-07
**41001**	N.S.	1.33E-06
**41254**	5.41E-07	1.04E-07
**41316**	7.43E-05	1.22E-06
**41524**	N.S.	9.13E-10
**41651**	N.S.	3.81E-06
**41668**	N.S.	1.83E-04
**41722**	N.S.	1.12E-04
**41728**	N.S.	1.32E-06
**41909**	8.88E-07	1.64E-10
**42299**	N.S.	3.86E-05
**42507**	1.62E-10	2.15E-05
**42837**	N.S.	4.29E-06
**42846**	9.50E-05	2.00E-07
**42953**	N.S.	7.72E-05
**43150**	2.83E-03	7.80E-09
**43199**	N.S.	2.62E-05
**43210**	N.S.	1.40E-06
**43226**	3.08E-07	1.58E-07
**43617**	N.S.	9.14E-05
**43959**	5.17E-03	7.33E-07
**44105**	2.13E-06	4.11E-06
**44235**	2.18E-07	1.13E-07
**44375**	N.S.	2.46E-07
**44572**	2.35E-02	2.97E-08
**44994**	8.79E-03	2.45E-05
**45084**	9.47E-04	1.26E-04
**45127**	N.S.	2.13E-05
**45489**	N.S.	1.55E-06
**45653**	N.S.	1.21E-05
**46052**	N.S.	7.22E-07
**46054**	1.01E-07	2.20E-07
**46257**	N.S.	7.94E-07
**46315**	N.S.	1.93E-04
**46326**	N.S.	2.32E-06
**46560**	6.58E-10	2.45E-05
**46836**	N.S.	3.62E-05
**46888**	N.S.	2.50E-10
**47018**	5.92E-04	9.14E-07
**47061**	N.S.	2.19E-09
**47219**	N.S.	6.75E-11
**47279**	4.53E-08	3.44E-05
**47670**	8.70E-08	1.02E-08
**47700**	N.S.	9.87E-12
**47888**	6.10E-08	4.46E-07
**48185**	N.S.	5.05E-07
**48190**	8.63E-07	1.00E-08
**48267**	N.S.	1.66E-08
**48401**	7.20E-12	2.18E-08
**48415**	4.51E-04	4.59E-05
**48484**	N.S.	3.54E-08
**48491**	N.S.	2.77E-07
**48591**	N.S.	3.23E-09
**48817**	N.S.	6.16E-07
**48825**	3.92E-07	2.61E-07
**48829**	1.94E-02	7.54E-08
**48845**	N.S.	2.00E-03
**49054**	N.S.	2.45E-07
**49172**	N.S.	6.92E-06
**49213**	N.S.	1.03E-05
**49289**	9.75E-08	3.78E-09
**49302**	N.S.	2.15E-05
**49321**	N.S.	1.96E-06
**49469**	N.S.	2.65E-06
**49580**	1.12E-05	6.36E-07
**49639**	4.13E-04	2.40E-08
**49703**	3.78E-04	2.75E-07
**49849**	2.76E-02	1.19E-08
**49931**	N.S.	2.66E-06
**49979**	N.S.	8.43E-07
**49995**	N.S.	3.77E-06
**50066**	3.27E-05	4.64E-07
**50081**	N.S.	2.97E-07

Table S2. Summary table for p values of α_{MST} and α_{Extra} in the most discriminating 1-cycles from the main analysis. All p values were false discovery rate (FDR) corrected; significance level was defined by a corrected $p < 0.05$. N.S.: not significant.
